# Molecular Weight and Monosaccharide Composition of *Astragalus* Polysaccharides

**DOI:** 10.3390/molecules13102408

**Published:** 2008-10-01

**Authors:** Du-Juan Xu, Quan Xia, Jia-Jia Wang, Pei-Pei Wang

**Affiliations:** 1 Department of Pharmacy, the First Affiliated Hospital of Anhui Medical University, Hefei, 230022. P. R. China; E-mail: xiaquan@ah163.com (Q. X.); 2 School of Pharmacy, Anhui Medical University, Hefei, 230032. P. R. China; E-mails: wjj1982211@sina.com (J-J. W.), wangpeipei2001@sina.com (P-P. W.)

**Keywords:** *Astragalus* polysaccharides, Isolation and Purification, Molecular Weight, Monosaccharide composition

## Abstract

Two polysaccharides (APS-I and APS-II) were isolated from the water extract of *Radix Astragali* and purified through ethanol precipitation, deproteination and by ion-exchange and gel-filtration chromatography. Their molecular weight was determined using high performance liquid chromatography and gel permeation chromatography (HPLC-GPC) and their monosaccharide composition was analyzed by TLC and HPLC methods, using a refractive index detector (RID) and an NH_2_ column. It was shown that APS-I consisted of arabinose and glucose and APS-II consisted of rhamnose, arabinose and glucose, in a molar ratio of 1:3.45 and 1:6.25:17.86, respectively. The molecular weights (Mw) of APS-I and APS-II were 1,699,100 Da and 1,197,600 Da, respectively.

## Introduction

*Radix Astragali* is the dried root of both *Astrugalus membrunaceus* (Fisch.) Bge. var. *mongholicus* (Bunge) Hsiao and *Astragalus membranaceus* (Fisch.) Bge., which belongs to the Fabaceae family. It is a crude drug widely used in Traditional Chinese Medicine for thousands of years to treat various renal diseases. Polysaccharides from *Radix Astragali* are a class of macromolecules that have been shown strong anti-tumor [1] and anti-glomerulonephritis activities [2]. Studies have also shown that *Astragalus* polysaccharides (APS) have an effect on nephrotic syndrome [3], possess immunopotentiatiive functions [4] and cause improvement of early diabetic nephropathy [5]. In the present study, we extracted, isolated and purified the polysaccharide components from *Radix Astagali* and determined their molecular weight as well as their monosaccharide composition so as to further investigate its mechanism of action.

## Results and Discussion

**Figure 1:**
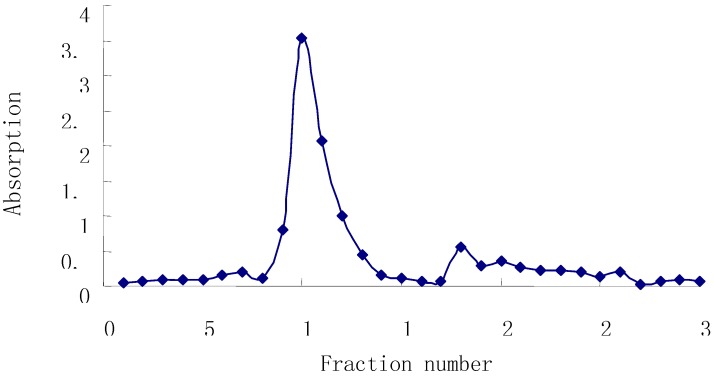
Elution Curve of *Astraglus* Polysaccharide on DEAE-52 Cellulose.

Though there are numerous literature reports on the extraction and purification of polysaccharides from *Radix Astragali*, only a few have studied the determination of its molecular weight and monosaccharide composition [6]. In the present work, the extraction and isolation of *Astragalus* polysaccharides was performanced by boiling-water decoction and ethanol precipitation to yield crude polysaccharide, then the Sevag method was used to remove protein components after re-dissolution of the crude polysaccharides. The solution was then dialyzed against distilled water for 2 days and precipitated by adding ethanol until the concentration of ethanol reached 80%. The precipitate was collected by centrifugation and washed successively with absolute ethanol and acetone to give a light yellow powder. For additional purification the *Astragalus* polysaccharides were subjected to DEAE-52 cellulose column chromatography, eluting with distilled water. Each 50 mL eluted fraction was collected and the content of sugar was monitored using the phenol-sulfuric acid method [7]. Fractions 8~14 were combined, concentrated to 100 mL and designated as the APS fraction. Figure 1 shows that one main peak was found in the elution trace by this method.

Then, this material was subjected to Sephadex G-150 column chromatography (2×60 cm), eluting with distilled water collecting 50 mL fractions with monitoring by the phenol-sulfuric acid method. Fractions 2~4 and 5~7 were combined, concentrated, and named APS-I and APS-II, respectively.

**Figure 2:**
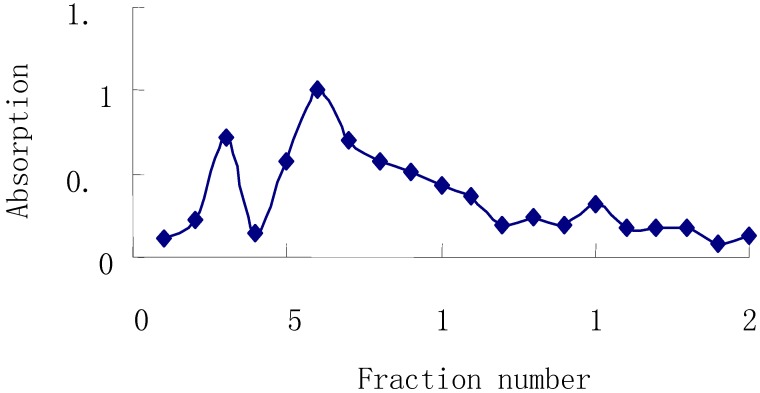
Elution Curve of APS on Sephadex G-150 column.

**Figure 3:**
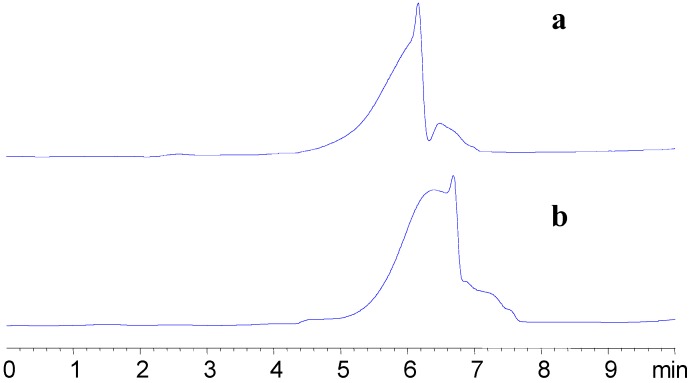
HPLC-GPC chromatography of APS-1 and APS-II.

A HPLC-GPC method was used to determine the molecular weight of APS-I and APS-II. Figure 3 shows the chromatograms of APS-I and APS-II. The molecular weight (Mw) of APS-I and APS-II, calculated using GPC software, were 1,699,100 and 1,197,600, respectively. The Dextran calibration curve of was Log (Mw) = 5.5324 – 0.4704 RT – 0.05829 RT^2^ (RT: retention time of Dextran).

Determination of the molecular weight of ASP-I and APS-II by HPLC-GPC run in isocratic mode. Mobile phase: 100% water; the column and optical unit of RID were set at 30°C and 35°C, respectively; flow rate, 1.0 mL/min; injection volume, 10 mL; (a) APS-I, retention time 6.162 min; (b) APS-II, retention time 6.397 min.

**Figure 4:**
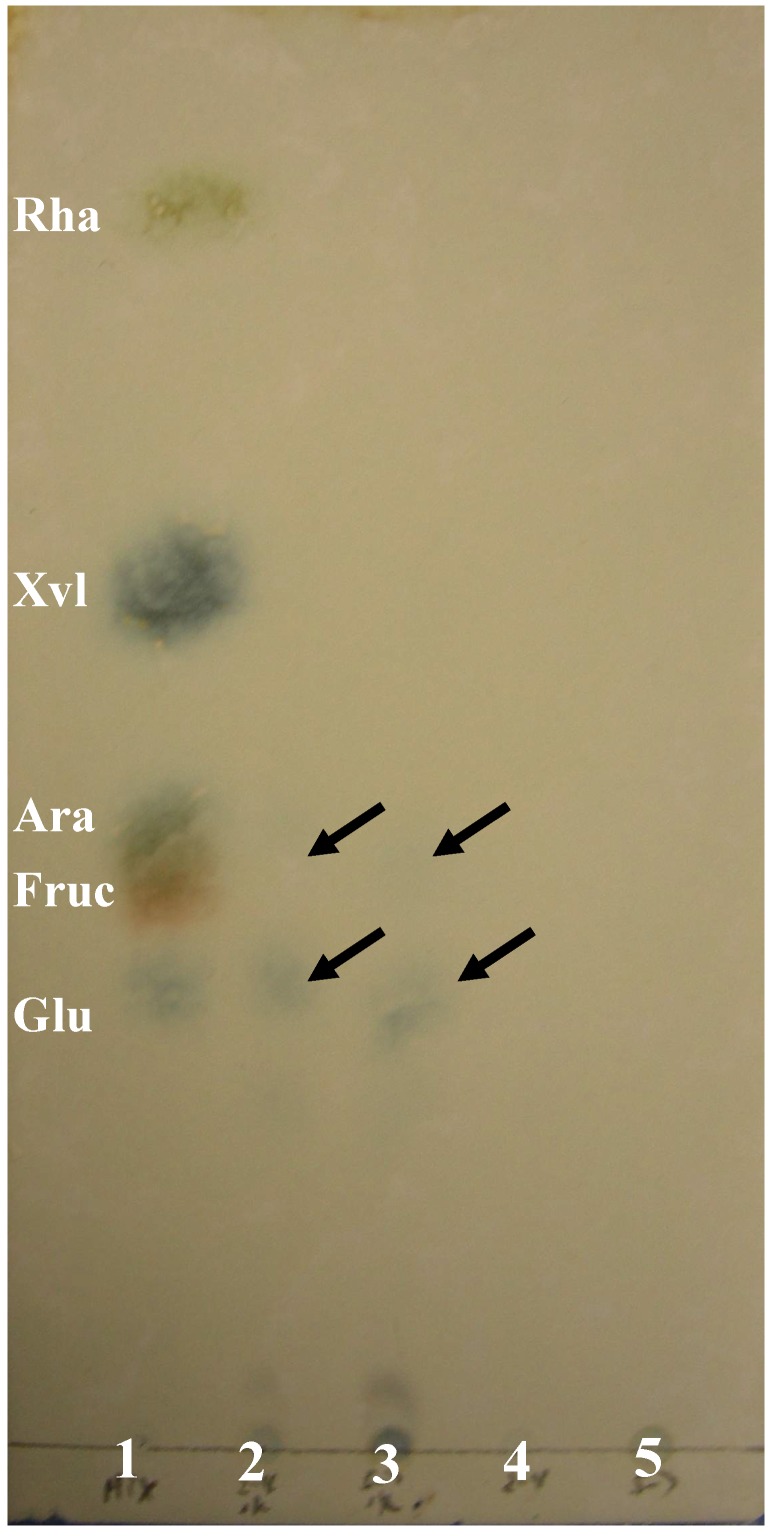
Monosaccharide composition analysis of APS-I and APS-II by TLC.

Monosaccharide composition analysis was carried out by TLC and HPLC methods. The TLC results presented in Figure 4 shows that the hydrolyzates of APS-I, APS-II and five mixed standard monosaccharides share the same arabinose and glucose spot.

Lane 1 correspond to five standard monosaccharides, Lanes 2 and 3 correspond to hydrolyzates of APS-I and and ASP-II, respectively, Lanes 4 and 5 correspond to aqueous solutions of APS-I and ASP-II, respectively.

**Figure 5:**
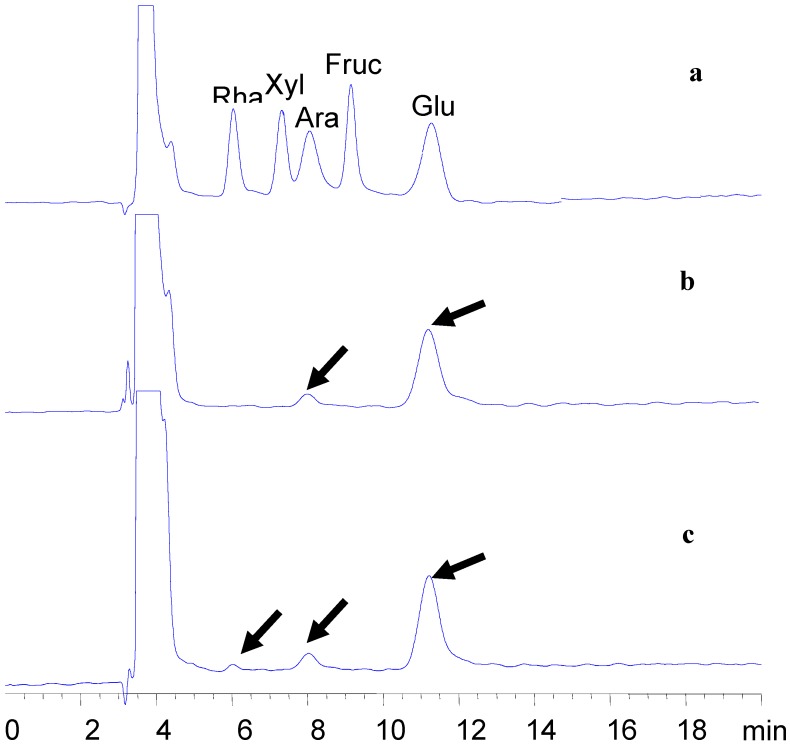
HPLC-RID analysis of monosaccharide composition.

The chromatogram in Figure 5 shows that using an HPLC-RID method, APS-I was composed of two monosaccharides – arabinose and glucose – in a molar ratio of 1:3.45; APS-II was composed of three monosaccharides – rhamnose, arabinose and glucose – in a molar ratio of 1:6.25:17.86.

(a) Mixed standard monosaccharides, rhamnose, xylose, arabinose, fructose and glucose, about 2 mg/mL for each monosaccharide. (b) APS-I hydrolyzates. (c) APS-II hydrolyzates.

## Experimental

### General

Standard monosaccharide (glucose, xylose, arabinose, rhamnose and fructose) and Dextran standards (12,000, 40,000, 70,000, 150,000, 500,000 and 2,000,000 Da) were purchased from Sigma. DEAE-Cellulose 52, Sephadex G-150, Sephadex G-200 and Sephacryl S-300HR were from Sinopharm Chemical Reagent. Co, Ltd. and Amersham Pharmacia Biotech, respectively. The polysaccharide content of the different eluting fractions was monitored using the phenol-sulfuric acid method, with an UV-Vis spectrophotometer (Beijing Purkinje General Instrument Co., Ltd., P.R. China).

### Plant material

*Radix Astragali* was purchased from Anhui GuoTou Chinese Medicine Co.Ltd. and authenticated by Professor Liu Shoujin, Department of Medicinal Plants, Anhui University of Traditional Chinese Medicine, as radix of *Astagalus membranaceus* (Fisch.) Bge. The *Radix Astragali* was cut into small slices (about 3 cm^2^ × 2 mm thick), kept in a vacuum drying oven at 60°C for 12 hours and then stored for further use.

### Preparation of extracts

Dried *Astragali* slices (100 g) were refluxed with petroleum ether (1 L) for 5 h to remove liposoluble constituents and then extracted with boiling water (1 L) for 5 h. The extraction process was repeated three times. The extracts were combined, filtered, concentrated and centrifuged. The Sevag method [8] was used to remove protein. The supernatant was added to absolute ethanol (4 vols) and kept overnight. The precipitate was collected and washed with absolute ethanol and acetone, then dried under vacuum, yielding a yellowish-brown crude *Astragalus* polysaccharide powder.

### Isolation and Purification

The crude polysaccharide was subjected to ion-exchange on a DEAE-52 cellulose column (4×60 cm), eluted with distilled water (1500 mL) and each 50 mL was collected with monitoring by the phenol-sulfuric acid method. The eluting fractions 8 to 14 were combined and concentrated to 100 mL, and named APS. The APS fraction was then subjected to gel-filtration chromatography on a Sephadex G-150 column (2 × 30 cm), eluting with distilled water (1000 mL) and collecting 50 mL fractions, using the same method to monitor the polysaccharide content. Fractions 2 to 4 and fractions 5 to 7 were combined and named APS-I and APS-II, respectively. Confirmation of the purity of APS-I and APS-II was done using a Sephacryl S-300HR column (1 × 30 cm) and a Sephadex G-200 column (1 × 30 cm).

### Determination of molecular weight

Dextran standards, APS-I and APS-II were dissolved in distilled water at a concentration of 2.0 mg/mL and analyzed on an Agilent 1100 Series HPLC system equipped with a RID and a TSK G5000PW_XL_ gel column (7.8 × 300 mm) and a TSK PW_XL_ (6.0 mm × 40 mm) guard column (TOSOH Corporation, Japan), to determine the retention time of standards and samples [9]. The column and detector compartment were maintained at 30°C and 35°C, respectively. Distilled water was used as mobile phase at a flow rate of 1.0 mL/min and injection volume was 10 μL. The molecular weight of APS-I and APS-II were calculated by constructing a calibration curve, in which the logarithm of the molecular weight of the Dextran standards ranging from 12,000 to 2,000,000 Da was plotted as a function of the retention time using Agilent ChemStation GPC Data Analysis Software (Rev. A.02.01).

### Monosaccharide composition analysis

APS-I and APS-II were hydrolyzed in 1 mol/L trifluoroacetic acid for 10 h at 100 °C in a sealed glass tube. The residual acid was removed under a stream of N_2_ in a 60°C water-bath after adding methanol and taking to dryness three times, then distilled water (1 mL) was added to dissolve the residue.

*TLC method* [10]: TLC glass plates (5 × 10 cm glass plates with 0.2 mm thick silica gel, QingDao Ocean Chemical Plant, P.R. China) were incubated overnight with 0.3 mol/L aqueous NaH_2_PO_4_ solution. The plates were then activated at 115°C for 1 h before use. Five standard monosaccharides and the hydrolyates of APS-I and APS-II (2 μL per spot) were applied on the TLC plates which were developed in TLC chambers. The first eluant [4:3:1 (v:v:v) *n*-butanol-acetone-water] was run to a height of 9 cm from the origin. After drying, the plates were developed again with the second eluent [8:4:7:3 (v:v:v:v) *n*-butanol-acetic ether-isopropanol-water] to a height of 9 cm from the origin. After drying, the plates were sprayed with 2% phenylamine - 2% diphenylamine - 85% phosphoric acid [dissolved in acetone in a ratio of 5:5:1 (v:v:v)] and then heated in oven at 105 °C for 1 h.

*HPLC-RID method* [11]: The hydrolyzates of APS-I and APS-II were analyzed by HPLC on an Agilent 1100 series HPLC (Agilent Technologies Palo, Alto, CA, USA) equipped with a RID, using an Hypersil NH_2_ column (4.6 × 250 mm, Dalian Elite Analytical Instrument Co. Ltd., P.R. China). The flow rate was 1 mL/min and the mobile phase was acetonitrile-water (82:18). The injection volume of mixed monosaccharide standards and APS-I and APS-II hydrolyzates was 10 μL. The temperature of column and optical unit were set at 30 °C and 35 °C, respectively. Identification of the monosaccharides in the hydrolyzates of APS-I and APS-II was carried out by comparing their retention times with those of standards under the same HPLC conditions. Quantitative determination was performed using the external standard method.
